# Health-related Sustainable Development Goals: countdown on alcohol use, smoking prevalence, child overweight and suicide mortality

**DOI:** 10.1093/eurpub/ckaa027

**Published:** 2020-05-11

**Authors:** Maaike Droogers, Danielle Jansen, Jutta Lindert, Luis Saboga-Nunes, Mathilda Rudén, Marie Guichardon, Dineke Zeegers Paget

**Affiliations:** c1 European Public Health Association (EUPHA), Utrecht, Netherlands; c2 Department of Health Sciences, University Medical Center Groningen, University of Groningen, Groningen, Netherlands; c3 Department of Health and Social Work, University of Applied Sciences Emden, Emden, Germany; c4 Women’s Research Center, Brandeis University, Waltham, MA, USA; c5 Institute of Sociology, University of Education Freiburg, Germany; c6 NOVA National School of Public Health, Public Health Research Centre, Universidade NOVA de Lisboa, Lisbon, Portugal; c7 Comprehensive Health Research Center (CHRC), Lisbon, Portugal; c8 Institute of Environmental Health (ISAMB), Lisbon, Portugal

## Abstract

The Sustainable Development Goals (SDGs) are a set of goals that aspire to ‘leave no one behind’, adopted by all members of the United Nations and to be achieved by 2030. Now, four years after the SDGs entered into force, we examine the progress towards the health-related SDGs in the European region. In this region, least progress is made towards the targets set for alcohol consumption, smoking prevalence, child overweight, and suicide mortality. For each of these challenges we take stock of current policies, continuing challenges, and ways forward. Written from the perspective of European Public Health Association (EUPHA) we emphasize the potential contribution of civil society organizations in attaining the health-related SDGs.

## Introduction

Many countries are failing to achieve the Sustainable Development Goals (SDGs), agreed by all members of the United Nations and which came into force on 1 January 2016. The Goals cover the major issues confronting humanity and the planet itself and include targets to be met by 2030. Progress towards these targets is monitored using 232 indicators, of which 52 can be related to our health. On current trends, not even the most advanced countries will achieve the health goals.^1^^,^^2^ Now, 4 years after the SDGs entered into force, we ask ourselves: how is Europe progressing towards the health-related SDGs?

We answer this question by examining the European data from Global Burden of Disease (GBD) Study, which gives a score for how well each country is progressing towards the health-related indicators.^1^ We present the four lowest scoring indicators by providing a short description of the issue, including current policy frameworks and initiatives in Europe.

## Lowest scoring health-related SDGs

An examination of the European data extracted from the GBD Study, which projected attainment on the basis of past trends of the health-related indicators in 195 countries from 1990 to 2017, shows that Europe is progressing well overall towards the health-related SDGs, compared to other regions (with an average score of 76.9 out of 100).^1^ In part, this is because some of the indicators relate to diseases that are rarely found in Europe or issues that have long been addressed, such as malaria incidence, proportion of births attended by skilled health personnel, and prevalence of neglected tropical diseases. However, there are also several indicators that risk not being achieved by 2030. The lowest scoring health-related indicators in Europe are: alcohol consumption, smoking prevalence, child overweight and suicide mortality (see table 1).

**Table 1:** Lowest scoring health-related SDG indicators in the European region, with 0 capturing the worst levels and 100 capturing the best levels

Indicator name	Indicator description	Median score in European region^a^ (out of 100)	Scoring range (out of 100)
Alcohol use	Indicator 3.5.2: risk-weighted prevalence of alcohol consumption, as measured by the summary exposure value for alcohol use	25.6	Huge scoring range, from 0.5 (Ukraine) to 85.2 (Turkey)
Smoking prevalence	Indicator 3.a.1: age-standardized prevalence of current smoking in populations aged 10 and older	35.9	Huge scoring range, from 3.5 (Montenegro) to 85.8 (Tajikistan)
Child overweight	Indicator 2.2.2b: prevalence of overweight in children aged 2–4 years	37.5	Huge scoring range, from 13.0 (Montenegro) to 85.8 (Moldova)
Suicide mortality	Indicator 3.4.2: age-standardized death rate due to self-harm, per 100 000 population	50.4	Huge scoring range, from 3.6 (Lithuania) to 95.1 Turkey

Data from GBD 2017.^1^

a As per WHO European region (*n*=51, not including Monaco and San Marino).

### No safe level of alcohol consumption

Target 3.5 of the SDGs reads ‘strengthen the prevention and treatment of substance abuse, including narcotic drug abuse and harmful use of alcohol’. This implies that there is a healthy level of alcohol use even though it is now known that no level of alcohol consumption can be considered safe.^3^

In 2016, consumption of alcohol resulted in some 3 million deaths (5.3% of all deaths) and 132.6 million disability-adjusted life years (DALYs) (5.1% of all DALYs), worldwide.^4^ Europe is the region in which people consume the most alcohol and with the highest levels of alcohol-related harm.^5^ The European action plan to reduce the harmful use of alcohol between 2012 and 2020, a policy endorsed by all countries in the European region, includes a variety of measures to tackle alcohol-related harm.^6^ These measures are similar to those that work best to reduce consumption of any harmful substance, such as nicotine products and junk food, and include:

1. Reducing affordability, e.g. through taxation or minimum price per unit.
2. Marketing restrictions.
3. Restricting physical availability of alcohol.^6^

The Nordic countries have been especially successful in this area, with the national alcoholic beverage retailing monopolies: Systembolaget in Sweden, Vinmonopolet in Norway and ALKO in Finland.^6^

### Smoking is still a public health issue

In the European Union (EU), 700 000 people die annually from the direct consequences of tobacco use.^7^ Europe is also the region with the highest prevalence of smokers.^8^ Despite overwhelming evidence of the negative effects of nicotine intake, the prevalence of smoking is not expected to decline in the near future.^8^

European Union policy is based on the Tobacco Products Directive (2014/40/EU) that became applicable in EU countries on 20 May 2016, supplemented by national regulations on plain packaging of tobacco products and smoke free public spaces. The European Directive lays down rules governing the manufacture, presentation and sale of tobacco and related products. The main global policy framework is the WHO Framework Convention on Tobacco Control (WHO FCTC).

Taxation of cigarettes is an effective measure to reduce tobacco use. For example, it has been shown in Europe that smoking consumption decreases by 5–7% for every 10% increase in the price of cigarettes.^9^ However, there is a growing threat from the aggressive marketing, in some countries, of electronic nicotine delivery systems and electronic non-nicotine delivery systems (ENDS/ENNDS) (e-cigarettes), which act as a gateway for young people by recruiting them to nicotine addiction^10^ and which add to the risks of smoking among those engaged in dual use^11^.

Regulations and policies on alcohol and tobacco are not a matter of the ‘nanny state’ intervening, rather these are to counterbalance the aggressive marketing and sales of tobacco and alcohol industry. These commercial determinants of health are a priority for non-governmental organizations (NGOs) to advocate against.^12^ The role of NGOs and civil society organizations in achieving the health-related SDGs is discussed later.

### Overweight in children

In 2018, 40 million children under 5 and 340 million aged 5–19 years were overweight or obese globally. If current trends continue this number will increase to 70 million by 2025.^13^ In European countries, the prevalence of overweight in children is still rapidly increasing or stabilizing at very high levels.^14^ Overweight children are at increased risk of developing cardiovascular disease, type 2 diabetes and hypertension later in life.

Despite strenuous attempts by corporations to blame reduced exercise, it is now clear that changing dietary trends are an important driver of childhood overweight, both globally and in Europe, although there are differences between urban and rural areas.^15^ There is some evidence that political stability and more effective governance are associated with less overweight as this provides more opportunities for policymakers to focus on key public health problems, such as obesity.^16^ Also the degree of regulation (e.g. consumer protections, lack of pricing freedom) contributes to a reduction of overweight children.^17^

To tackle the issue of childhood overweight and obesity, policies have been developed at multiple levels. Examples of important ‘upstream’ policies (aimed at influencing underlying determinants of health in society) are the EU Action Plan on Childhood Obesity^18^ and the White Paper on a Strategy for Europe on Nutrition, Overweight and Obesity-related Health issues^19^, adopted by the European Commission. Both initiatives emphasize a shared and integrated EU approach in reducing childhood overweight and obesity by providing priority areas for action on both community and national level regarding, e.g. change of food availability and physical activity environment. ‘Midstream’ policies (aimed at directly influencing population behaviours) concern mainly national or local interventions that take place in school settings focussing on preventing or managing weight gain in children and adolescents. A widely known and supported example is the WHO’s initiative on Health Promoting School^20^ which entails a holistic approach to promoting health in schools. ‘Downstream’ policies are often individual-based policies and refer to the support-health services provide to manage and reduce existing overweight problems in children, such as strategies to change diet or activity levels in children. A combination of policies at different levels—multicomponent interventions—are more likely to be associated with positive results.^21^

No European government has yet implemented a comprehensive set of policy approaches that are effective in reducing childhood overweight.^22^ Future actions should focus on multicomponent interventions including policies at different levels, different methods, and involvement of other stakeholders besides governments, such as industry and civil society.^22^ Examples of civil society engaged in this issue include the FDI World Dental Federation and the International Baby Food Action Network, which promotes breastfeeding as a way to reduce the risk of childhood overweight and obesity. Although it is clear that civil society should have an important role in combatting childhood overweight and obesity, it is not yet clear in what way these NGOs are supposed to act and how to deal with conflict of interests.

### Suicide

Suicide is a serious, potentially preventable cause of premature death.^23^ Globally, at least 800 000 individuals die by suicide every year.^24^ However, precise global estimates are difficult to obtain, as only 15% of WHO Members States have comprehensive vital data registration and underreporting and misattribution is common. In the past 50 years suicide rates—or knowledge of suicide rates—has increased significantly (approximately by 60%) despite prevention efforts.^25^ In 2016, the global age-standardized suicide rate was 10.5/100 000. In the European region the highest ‘official’ rates of suicide mortality are reported in Lithuania (age-standardized rate: 27.4/100 000) and the lowest ‘official’ rates are reported in Turkey (age-standardized rate: 3.2/100 000).^1^ International commitments strive to reduce these numbers. The WHO’s World Mental Health Action Plan sets a target of 10% reduction in countries’ annual mortality rates due to suicide by 2020. In addition, the SDG target is to reduce suicide mortality rate by one-third.

Suicide rates vary between and within countries. Most suicides occur in middle- and low-income countries (∼70%). In high-income countries, suicide is most common among middle-aged and elderly men and among 15- to 20-year olds.^26^ Suicide mortality is unevenly distributed by country, gender, age group, socioeconomic status and time of the year.^27^ Based on this heterogeneity, several risk factors for suicide mortality have been identified and classified as primary (e.g. presence of psychiatric and medical conditions), secondary (e.g. adverse life events and situation) and tertiary (demographic factors, such as male gender and old age).^28^ Primary factors affect almost everybody: 90% of individuals who died by suicide had a psychiatric disorder, such as major depressive episodes, bipolar disorders, borderline or antisocial personality disorder.

Considering the high prevalence of suicide mortality, suicide prevention is of prime importance. Suicides are preventable but unfortunately suicide prevention is often a low priority for governments and policymakers. In 2014, the WHO published its first-ever world suicide prevention report.^29^ Following this report, the number of countries with national suicide prevention strategies has increased but with the total number of 38 countries with strategies is still few.

European countries have developed a number of measures, such as reducing access to means of suicide, school-based interventions, introducing alcohol and substance abuse policies and public education campaigns to improve recognition of suicide risk and help seeking. However, studies suggest modest effects of public education and awareness campaigns. It has been suggested that community networking is effective in reducing suicides, while awareness programmes, training of gatekeepers and other ‘educational-type’ campaigns have limited long-term effects.^30^^,^^31^

Suicide prevention research needs to be prioritized on the global public health agenda. Good practice examples of interventions should be systematically mapped and analysed. One of these might be the Cross-Government suicide prevention strategy for England. This strategy emphasizes cross-sectoral collaboration across national government, its agencies and voluntary and charitable organizations. However, those at risk of suicide are changing: The highest rates in 2019 include middle-aged men and young people. Therefore, the set of actions to tackle risk factors for changing high-risk groups should be based on emerging priorities and reliance on up-to-date data. An understanding of protective factors may inform development of novel intervention strategies. Despite the amount of research done in the past decades on suicide mortality, the ‘cause of causes’ remains unknown.

## Discussion

Health is an integral part of the SDGs, with health-related indicators spread across nearly all 17 goals.^32^ In this article, we highlighted the health-related indicators that, based on the projections of the GBD Study^1^, have the least chance of being attained. In 2020, only 10 more years are left to achieve the SDGs (the GBD study has a data visualizer that allows to explore the health-related SDGs data in great detail. The tool is available at https://vizhub.healthdata.org/sdg/).

It is not only heads of state that are committed to the SDGs but also civil society. There are numerous ways in which civil society can contribute to achieving the health-related SDG targets. For example, civil society organizations can play an important role in holding governments accountable for the goals. Despite the non-binding nature of the SDGs, the goals can be linked to existing international legally binding human rights laws.^33^ Identifying these laws can assist civil society in demanding governments to take accountability for the SDGs. The SDGs are sometimes perceived as more global issues, potentially hindering the action in communities and by regional authorities. Civil society organizations can translate of the SDGs to local action. Influencing national and local policies requires formulating the issues in such a way that they resonate with local decision makers and policymakers. Conversely, civil society organizations can also play a role in translating local and national policies and practices into global terminology, to demonstrate that local action is consistent with global commitments. And science-based societies can help identify the determinants on which action can be based. The benefits of civil society engagement in the health arena are plentiful, including empowerment, service delivery, commitment, flexibility, participation in policy and credibility.^34^

We recognize that the data used for identifying the lowest scoring health-related indicators in Europe have some limitations. The GBD Study describes numerous limitations, including indicator measurement, forecasting and attainment analyses, indicator scaling and index construction and data availability and disaggregation. Nevertheless, the scoring of the indicators allows ranking the health-related indicators and identifying those that risk not being achieved. In the future, to improve our understanding of the burden of disease, better data are needed, including primary data from original studies and secondary data from other sources, e.g. Organization for Economic Co-operation and Development based data.

Despite the limitations of the data, it is clear that action is needed. Taken together, the lowest scoring indicators might be related to exposures to traumatic events. We are increasingly aware that substance abuse, such as alcohol and nicotine consumption, overweight, obesity and suicidal behaviour are related to traumatic experiences. How can there be more awareness about this relationship that has long-lasting and far-reaching effects with diverse outcomes? This should be on the agenda over the next few years if we are to attain the health-related SDGs in the European region.

*Conflicts of interest*: None declared.
